# In Vivo Subacute Toxicity and Antidiabetic Effect of Aqueous Extract of* Nigella sativa*

**DOI:** 10.1155/2017/8427034

**Published:** 2017-12-12

**Authors:** Karima Bensiameur-Touati, Ghouti Kacimi, El-Mehdi Haffaf, Sihem Berdja, Souhila Aouichat-Bouguerra

**Affiliations:** ^1^Laboratory of Physiology of Organisms, Team of Cellular and Molecular Physiopathology, Faculty of Biological Sciences, University of Sciences and Technology Houari Boumediene, BP 32, EL Alia, 16011 Algiers, Algeria; ^2^Laboratory of Biochemistry, Central Hospital of Army, Ain Naadja, 16005 Algiers, Algeria; ^3^Laboratory of Nuclear Medicine, Central Hospital of Army, Ain Naadja, 16005 Algiers, Algeria

## Abstract

*Context. Nigella sativa* seeds are usually used as traditional medicine for a wide range of therapeutic purposes.* Objective.* To investigate the subacute toxicity of NS aqueous extract and select its lowest dose to study its antidiabetic effect.* Methods.* 5 AqE.NS doses (2, 6.4, 21, 33, and 60 g/Kg) were daily administered to mice by gavage. Biochemical parameters measurements and histological study of the liver and the kidney were performed after 6 weeks of supplementation. Thereafter, and after inducing diabetes by alloxan, rats were treated by 2 g/Kg of AqE.NS during 8 weeks. Metabolic parameters were measured on sera. A horizontal electrophoresis of plasmatic lipoprotein was conducted. Glycogen, total lipids, and triglycerides were measured in the liver. TBARS were evaluated on adipose tissue, liver, and pancreas.* Results.* AqE.NS showed no variation in urea and albumin at the 5 doses, but hepatotoxicity from 21 g/Kg was confirmed by histopathological observations of the liver. In diabetic rats, AqE.NS significantly decreased glycemia, TG, T-cholesterol, LDL-c, and TBARS and showed a restored insulinemia and a significant increase in HDL-c. Results on the liver indicated a decrease in lipids and a possible glycogenogenesis.* Conclusion.* AqE.NS showed its safety at low doses and its evident antihyperglycemic, antihyperlipidemic, and antioxidant effect.

## 1. Introduction

Diabetes mellitus is one of the most common lifestyle diseases. It is a major endocrine disorder that is among the most important clinical risk factors involved in several disorders, including atherosclerosis, neuropathy, nephropathy, retinopathy, and stroke.

The resulting metabolic disorders are characterized by hyperglycemia resulting from defects in insulin secretion and/or insulin action and by altered metabolism of lipids, carbohydrates, and proteins [1, 2].

Due to its high prevalence (6.4% of the population (i.e., 285 million people), with an expected increase in the adult population by 2030 estimated to be 370 million by the World Health Organization) and the physical, psychological, and social impact it has, diabetes is considered as a major medical concern.

Among all types of diabetes, type 2 diabetes is the most complicated. Generally, current therapeutic strategies for this type of diabetes are limited and involve insulin and four main classes of oral antidiabetic agents that stimulate pancreatic insulin secretion (sulphonylureas and rapid-acting secretagogues/insulinotropics), reduce hepatic glucose production (biguanides), delay digestion and absorption of intestinal carbohydrate (*α*-glucosidase inhibitors), or improve insulin action [thiazolidinediones (TZDs)] [2].

Each of the above agents suffers generally from inadequate efficacy and a number of serious adverse effects [3]. Therefore, there is a need to develop safe and effective treatment modalities for diabetes. In this regard, plants are specifically reservoirs of bioactive compounds as they synthesize complex organic molecules for their structures and functions [4], so herbal medicines are becoming an important component of the trend towards alternative medicines.

Elsewhere, various plants have been found to possess significant antidiabetic property after their preclinical and clinical evaluation [5].

*Nigella sativa* L., a dicotyledonous plant belonging to the Ranunculaceae family, is among the intensively researched medicinal plants [6]. It has a rich historical and religious background [7]. It is the black seed referred to by the prophet Mohammed as having healing powers, also identified as the curative black cumin in the Holy Bible, and is described as the Melanthion of Hippocrates and Dioscorides and as the Gith of Pliny.

It is an annual herb native from the Mediterranean area and is also found to be growing in some other regions in the world such as Saudi Arabia, Syria, the Middle East, and North Africa and has also been widely cultivated throughout Southern Europe, Asia, Turkey, Pakistan, and India [7, 8].

Seeds of* N. sativa,* which are commonly known as black seeds or black cumin, have several traditional uses as spice, carminative, condiment, and aromatic [9] and are eaten with honey, sweet foods, bread, and cheese. They are also considered as the source of the active ingredients of this plant.

The studies about the composition of* N. sativa* have demonstrated that its seeds are a complex substance made of more than 100 compounds, some of which have not yet been identified or studied [10]. We actually know that seeds contain fixed oils and volatile oils. They are a rich source of quinones, in particular thymoquinone (TQ), to which the significant activity of seeds is attributed and which was purified by El-Dakhakhny [11]. They also contain unsaturated fatty acids, amino acids, proteins, traces of alkaloids (nigelline, nigellimine, and nigellicine), and terpenoids.

Most of the studies on the biological effects of* N. sativa* have dealt with its crude extracts in water or different solvents; however, some studies examined also its active principles, especially TQ. In some reviews [7, 8, 12],* N. sativa* is reported to possess antitumor, antidiabetic, cardiovascular, pulmonary protective, antiasthmatic, gastroprotective, antifertility, diuretic, antispasmolytic, anti-inflammatory and analgesic, antimicrobial and antifungal, antioxidant, anticonvulsant, antinociceptive, antiurolithatic, neuroprotective, anxiolytic, nephroprotective, hepatoprotective, immunomodulatory, and anthelmintic activities, but a number of them are yet to be explored.

In our study, we were particularly interested in the aqueous extract of the seeds of this plant. We first studied the subacute toxicity of the aqueous extract in mice seeking safety and how toxicity can be expressed when present; and next we prospected the antidiabetic and antioxidant effects of this extract in rats.

## 2. Materials and Methods

### 2.1. Plant Materiel and Preparation of Aqueous Extract of* Nigella sativa*

*Nigella sativa* seeds used in this study come from Adrar, a region of southern Algeria.

Seeds were identified by Dr. Nabila Amirouche, the botanist from Faculty of Biological Sciences, University of Sciences and Technology “Houari Boumediene.”

Aqueous extraction was performed by a slightly modified method of Vahdati-Mashhadian et al. [13]: 150 g of black seed powder was mixed and then added to 500 ml of water and left for 12 h at 95°C on a thermostated stirrer. The extract was filtered through a muslin and the filtrate was transferred to a glass Petri dish and left at 90°C for 8 h until obtaining a pasty extract that was left at 4°C before use. The extraction yield is on average 24%.

### 2.2. Animals and Experimental Design Dietary Treatments

The study of the subacute toxicity of the aqueous extract of* Nigella sativa* seeds was carried out during 6 weeks on (6–8-week-old)* Mus musculus* mice with an average weight of 29.84 g by daily gavage using an oesophageal probe.

Mice were obtained from the Pasteur Institute of Algeria. The animals were fed on a standard pellet diet and water ad libitum. They were maintained in a controlled environment under standard conditions of temperature and humidity with an alternating 12/12 h light/dark cycle.

They were kept under fasting conditions 12 h before the gavage.

30 young female mice (more sensitive than males according to the Organisation for Economic Cooperation and Development: OECD) [14] that were healthy, nulliparous, and nonpregnant were randomly separated in 6 groups of 5 mice each: a control group that received distilled water and five groups corresponding, respectively, to the doses studied: 2 g/kg, 6.4 g/kg, 21 g/kg, 33 g/kg, and 60 g/kg of body weight.

During gavage, concentrations are calculated such that the maximum volume administered to mice does not exceed 0.3 mL.

The animals were under close observation during 24 h with a special attention immediately after gavage and during the first four hours.

The effect of the AqE.NS on animals' weight was recorded each week.

The study of the antidiabetic effect of the AqE.NS was carried out on female* Rattus norvegicus*, Wistar variety, with an average weight of 196.5 g. Diabetes was induced by a single intraperitoneal injection of alloxan (ALX) at a concentration of 200 mg/kg of body weight.

After 3 weeks, the effective introduction of diabetes was confirmed by the blood glucose test (>120 mg/dL). Therefore, 3 final groups of 5 rats each were constituted: a control group (Group C) of non-ALX and untreated healthy rats, a group of diabetic control rats or diabetic model (DM), and a group of diabetic rats treated with aqueous extract of* Nigella sativa* (DNS) at a dose of 2 g/kg (dose retained following the results of the subacute toxicity study). The antidiabetic effect was studied during 8 weeks by daily gavage using an oesophageal probe. The control group received distilled water.

The body weight of animals was recorded each week.

### 2.3. Methods

#### 2.3.1. Biochemical Analysis

*(1) Serum Assays*

*(i) Toxicity Markers.* After the 6 weeks of the experiment, the animals were bled from the retroorbital venous plexus; this technique replaces the use of anaesthetic agents, which affect the measurements of biochemical parameters. Blood, which is collected in dried tubes, was centrifuged at 3000 rpm for 10 min and the sera of each group were pooled in order to obtain a sufficient volume of serum to carry out the different dosages. Sera were stored at −20°C before use. Plasma urea, creatine, albumin, and hepatic function, aspartate transaminase (AST), alanine transaminase (ALT), and alkaline phosphatase (AP), were measured by the automated COBAS-INTEGRA 600 PLC.

*(ii) Biochemical Parameters for Antidiabetic Study.* For the study of the antidiabetic effect of the AqE.NS, rats were bled weekly by retroorbital puncture in order to dose glucose, triglycerides (TG), and total cholesterol (T-cholesterol) using Biosystems kits, AST, ALT, high-density lipoprotein-cholesterol (HDL-c), and low-density lipoprotein-cholesterol (LDL-c) by the automated COBAS-INTEGRA 400 controller, and insulinemia by radioimmunoassay using CIS test kit (ORIS INDUS), and the detection of lipoprotein was also performed by horizontal electrophoresis on agarose gel (1%) according to Kawakami method [15].

*(iii) Serum and Erythrocytic TBARS.* The antioxidant effect was assessed by the evaluation of serum and erythrocytic MDA, an end product of fatty acid peroxidation which reacts with TBA (thiobarbituric acid) to form TBARS (thiobarbituric reactive substances), a colored complex that has maximum absorbance at 532 nm.

After separation of plasma, the packed erythrocytes were washed with phosphate-buffered saline (PBS 1x) and lysed with buffer lysis (0.1 g of 0.1 M HCl Tris in 10 ml of H_2_Od at pH 6.6 added to 500 *μ*l of 0.5% triton X100); then the serum and the erythrocytes lysate MDA were, respectively, measured according to the method of Yagi [16].

*(2) Tissue Assays.* At the end of the study period, animals were sacrificed after urethane anaesthesia (0.4 mL/100 g). The liver and pancreas collected allowed evaluating glycogen, TBARS (MDA), total lipids, and tissue triglycerides. MDA was also assessed on adipose tissue.

*(i) Hepatic Glycogen.* The principle here was to hydrolyse hepatic glycogen extracted from liver into glucose which will be measured by the Folin and Wu method [17].

Concentrations were deduced from a standard curve prepared with standard glucose solution and the amount of glycogen is expressed per 100 g of fresh tissue.

*(ii) Total Lipids and Triglycerides.* The total lipids were evaluated according to the method of Folch et al. [18] and estimated in mg/100 g of tissue.

Triglycerides were measured from previous filtrates used for the quantification of total lipids (obtained after liver grinding, filtration, and adjustment with Folch), using Biosystems kits, according to the method of Wahlfeld and Bergmeyer [19].

*(iii) Tissue TBARS.* For this purpose, liver, pancreas, and adipose tissue were ground in liquid nitrogen and the lipid peroxidation was evaluated on the resulting crusts by the TBARS assay according to the method of Ohkawa et al. [20] related to evaluation of lipid peroxides in animal tissue.

Results were estimated by the absorbance at 532 nm and expressed in micromoles of MDA/100 g of organ.

#### 2.3.2. Histological Study

At the end of the experimental study of the toxicity effect of AqE.NS and after sacrificing animals with 0.4 ml of urethane (25%)/100 g of body weight, the livers and kidneys were harvested and fixed in paraformaldehyde (4%) for 24 h. After washing the samples during 2 h under a thin stream of running water, the fragments were stored in isopropanol (70%) before their dehydration in successive ethanol baths at increasing concentration and their embedding in paraffin. Samples were thereafter cut at 2 *μ*m and stained with Masson's trichrome [21]. The cuts were observed under a light microscopy with a camera system (HIROCAM.MA.88-500).

### 2.4. Statistical Study

Data were expressed as means ± standard error of means (SEM). A Pearson's Chi-square test (*χ*^2^) was performed to test the null hypothesis that no association exists between the doses of AqE.NS and the toxicity markers and all antidiabetic parameters were tested for significant differences between groups of rats and effect of treatment using one-way and two-way ANOVA followed by Tukey's post hoc test, using R Commander {Rcmdr} statistical package. The differences at level < 0.05 were considered to be statistically significant.

## 3. Results

### 3.1. AqE.NS's Subacute Toxicity

As shown in Table 1, the study of the subacute toxicity of the AqE.NS administered daily to mice during 6 weeks leads to 1 death after 2 weeks of treatment with 6.4 g/Kg of AqE.NS, to 2 deaths after gavage during 5 weeks by the dose of 21 g/Kg, and to 3 deaths, respectively, observed after 3, 4, and 5 weeks, when mice were treated with 60 g/Kg. No other deaths have been recorded for the application of other doses.

**Figure 1:**
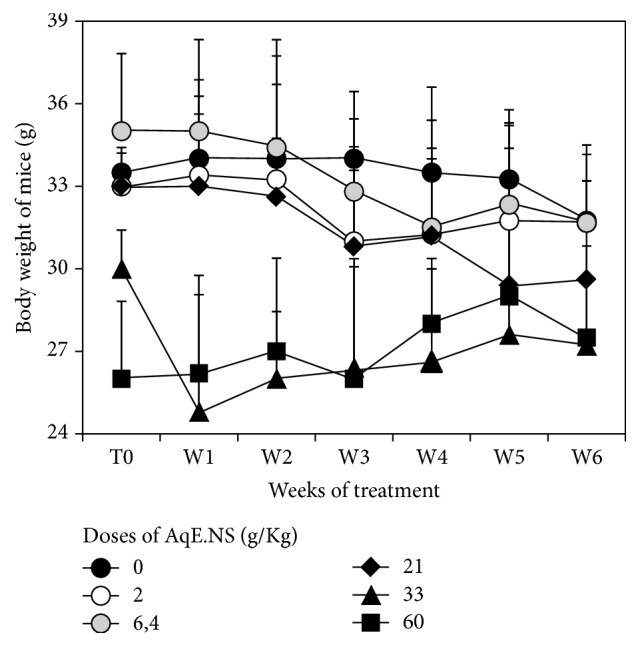
Variation of body weight of mice after gavage at different doses of AqE.NS.

Otherwise, we did not detect any correlation which was found between the body weights of the animals and the administered doses during the period of the experimental study (Figure 1).

Pearson's Chi-square test (*χ*^2^) (Table 2) shows that the increase of AqE.NS doses administered to rats induces a significant variation in creatine (*p* < 0.05), AST (*p* < 0.001), ALT (*p* < 0.001), and AP (*p* < 0.05) compared with control rats, while the urea and albumin did not differ between control rats and those used for treatment (*p* > 0.05).

**Figure 2:**
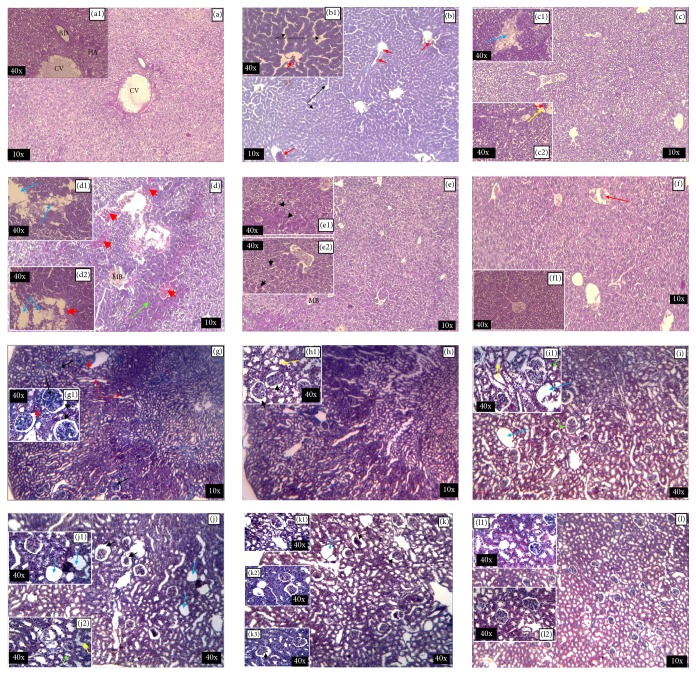
Effect of AqE.NS on morphological features of mice's liver and kidney observed using optic microscopy. ((a) G ×10, (a1) G ×40): control. ((b) G ×10, (b1) G ×40): 2 g/kg. ((c) G ×10, (c1) and (c2) G ×40): 6.4 g/kg. ((d) G ×10, (d1) and (d2) G ×40): 21 g/kg. ((e) G ×10, (e1) and (e2) G ×40): 33 g/kg. ((f) G ×10, (f1) G ×40): 60 g/kg. CV: centrilobular vein; HA: hepatic artery; BD: bile duct; MB: Mallory body; blue arrow: necrotic zone; black arrow: dilatation of sinusoids; red arrow: richness of vascularization; green arrow: fibrosis zone; yellow arrow: inflammatory zone; red arrowhead: hemorrhagic zone; black arrowhead: round cells with large nuclei. ((g) Gr ×10, (g1) Gr ×40): control. ((h) G ×10, (h1) G ×40): 2 g/kg. ((i) G ×10, (i1) G ×40): 6.4 g/kg. ((j) G ×10, (j1) and (j2): G ×40): 21 g/kg. ((k) G ×10, (k1), (k2), and (k3) G ×40): 33 g/Kg. ((l) G ×10, (l1) and (l2) G ×40): 60 g/Kg. Black arrow: glomeruli; red arrow: collecting tubules; blue arrow: glomeruli removed from their vascular pellet; green arrow: distal convoluted tubule; yellow arrow: proximal convoluted tubule; black arrowhead: expanded Bowman space; red arrowhead: blood vessel. M: medulla; C: cortex.

The histological observations of the liver control sections (Figure 2(a), (a1)) show the arranged and homogeneous cells, without any irregularity. Indeed, the hepatic parenchyma is microscopically recognized by the centrilobular veins towards which cords of the hepatocytes converge.

After 6 weeks of administration of 2 g/kg of AqE.NS (Figure 2(b), (b1)), the liver shows the sinusoidal expansion, the beginning of inflammation rating by the recruitment of inflammatory cells, and vascularization richness, but the cell's form remains normal.

The 6.4 g/kg dose caused hypervascularization to the liver tissue and increased the dilatation of sinusoidal capillaries. We have also noticed the persistence of inflammation and the appearance of necrotic areas (Figure 2(c), (c1), (c2)).

The 21 g/kg dose induces total disorganization of the hepatic tissue with the appearance of haemorrhagic zones, fibrosis zones, Mallory body, inflammatory zones, and clear necrosis (Figure 2(d), (d1), (d2)).

However, surprisingly, we found a return to the normal cellular organization in the rats receiving 33 g/kg by the observation of the form of rounded cells with a large nucleus, expressing resumption of cellular activity. Thus, the deep morphological disturbances observed previously fade; however, some alterations persist including dilatation of sinusoids, inflammation, and presence of Mallory's bodies (Figure 2(e), (e1), (e2)).

For the highest dose, 60 g/kg, it would seem that if the animal does not die, it adapts because we have observed very organized tissue recalling the architectonic in the control animal. The liver tissue seems to recover its functionality and the vascularization was normal. We notice though the presence of some inflammatory cells (Figure 2(f), (f1)).

Concerning the histological observation of kidney, the medulla and the cortex of the control group (Figure 2(g), (g1)) showed a regular structure of the glomeruli, the medulla, and the renal tubules. However, the morphological changes were observed in the mice used for treatment, particularly an increase in the Bowman space and the proximal lumen and the distal tubules at the doses of 2 g/kg (Figure 2(h), (h1)) and 6.4 g/Kg (Figure 2(i), (i1)). Also, some glomeruli were evacuated from their vascular pellets, leading to atrophied glomeruli at doses of 21 g/kg and 33 g/kg (Figures 2(j), (j1), (j2) and 2(k), (k1), (k2), and (k3)), whereas no histopathological changes were observed in the kidney sections of the treated mice at 60 g/kg (Figure 2(l), (l1), (l2)).

### 3.2. AqE.NS's Antidiabetic Effect

The alloxan injection of 200 mg/kg to rats allowed the introduction of diabetes with modifying some biochemical parameters, so significant differences were observed between the 3 groups of rats being studied, especially with glycemia (*p* < 0.001), TG (*p* = 0.01), T-cholesterol (*p* < 0.05), HDL-c (*p* < 0.01), LDL-c (*p* < 0.001), and ALT (*p* < 0.001) as shown in Tables 3 and 4, whereas for the insulin, ANOVA does not show any significant differences (*p* = 0.749), although a decrease was observed in Table 3 in the rats given alloxan: DM (29.41 ± 7.55 UI) and DNS (24.85 ± 4.60 UI) groups compared to the control (31.99 ± 4.17 UI).

However, glycemia (*p* < 0.001), TG (*p* < 0.01), T-cholesterol (*p* < 0.05), HDL-c (*p* < 0.01), LDL-c (*p* < 0.05), and also insulin level (*p* < 0.05) were significantly enhanced over time by the treatment with AqE.NS as shown in Table 4.

**Figure 3:**
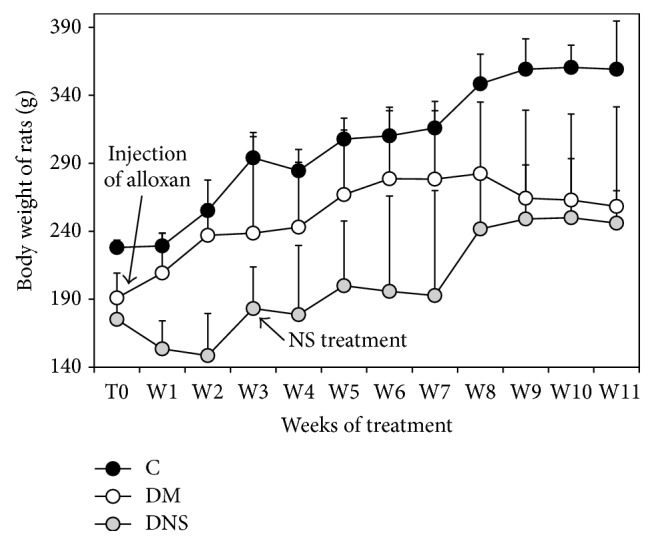
Variation of body weight of* Rattus norvegicus* treated with 2 g AqE.NS/kg during 8 weeks.

The weight's evolution curves show the same model for the three groups being studied (Figure 3), especially between groups C and DNS.

Also, Table 5 shows the significant difference between the weights of the three groups of rats (*p* < 0.0001), according to the duration of the experiment (*p* < 0.0001), but Figure 3 shows mainly a weight gain in the DNS group during the last weeks of treatment unlike the DM group.

**Figure 4:**
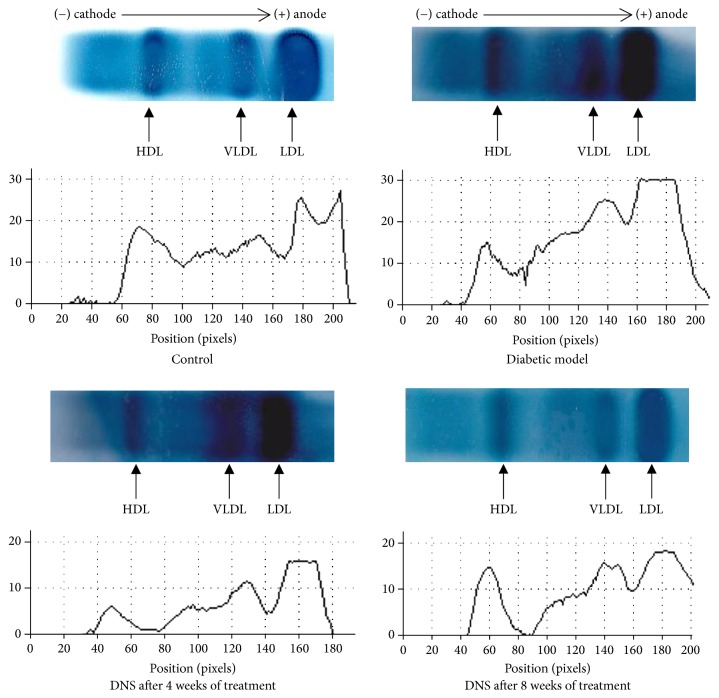
Profile of plasma lipoproteins by horizontal agarose gel electrophoresis on C, DM, and DNS rats after 4 and 8 weeks of treatment with 2 g/kg of AqE.NS.

The profile of plasma lipoproteins by horizontal agarose gel electrophoresis (Figure 4) shows in the control rat that VLDL predominated followed by LDL and then HDL, whereas in diabetic rats, VLDL and LDL increased in parallel with a decrease of HDL, while with the rats treated by AqE.NS, we observed a decrease in LDL and VLDL levels after 1 month of treatment, followed by an increase in HDL after two months of treatment.

If the Tukey test does not show the significant difference (Table 4) between the three groups of rats for the serum and erythrocytic TBARS after the injection of alloxan, we nevertheless note in Table 6, especially for the serum TBARS, an increase of 43% in the DM group ranging from 5.63 ± 1.31 to 9.85 ± 2.33 *μ*M/L and of 46% in the DNS group ranging from 4.35 ± 0.59 to 8.15 ± 0.34 *μ*M/L (Table 3). Furthermore, treatment with AqE.NS decreases the serum TBARS by about 40% after 8 weeks of treatment from 8.15 ± 0.34 to 4.94 ± 0.14 *μ*M/L.

These same markers of antioxidant activity analyzed in the tissues (Table 6) after induction of diabetes show an increase, though not significant (*p* = 0.073), of 30.66% in the TBARS in the pancreas which appeared to be the preferred target of alloxan (158.63 ± 9.8 in DM group versus 121.41 ± 8.69 *μ*M/100 g of tissue in C group). Any significant changes were observed in the liver and in the adipose tissue. The administration of AqE.NS to diabetic rats causes a nonsignificant but nonetheless visible decrease of TBARS both in the pancreas (139.52 ± 12.99 versus 158.63 ± 9.8 *μ*M/100 g of tissue) and in the liver (152.1 ± 12.01 versus 169.59 ± 27 *μ*M/100 g of tissue).

The other biochemical parameters evaluated in the liver (Table 7) show a significant increase in the total lipids after diabetes inducing (*p* < 0.05) but no significant changes in glycogen and TG. The treatment with AqE.NS reduced significantly the total lipids (*p* < 0.05) and not significantly TG (318.57 ± 36.4 versus 400.88 ± 36.44 mg/100 g of tissue, however, with a rate of 20.53%) and increases slightly glycogen (1653 ± 191.6 versus 1318.57 ± 126.11 mg/100 g of tissue with a rate of 25.36%).

## 4. Discussion

### 4.1. Subacute Toxicity of AqE.NS on Mice

The evolution of the body weight was relatively constant for all animals and did not appear to correlate with the dose. These observations join those of Benhaddou-Andaloussi et al. [22] and Vahdati-Mashhadian et al. [13] who did not observe the weight variation in the presence of the aqueous extract.

As the kidney is the highest important organ in the pathway elimination of the toxic substances, urea, creatine, and albumin were measured for detecting possible renal dysfunction caused by AqE.NS.

Our results show that the different AqE.NS doses did not induce any significant variation in serum urea and albumin concentrations, which corresponds to the results of Dollah et al. [23], which did not report any changes after oral administration to rats upon very low doses, 0.01, 0.1, and 1 g of NS seeds/Kg, and Badary et al. [24], who assessed the effect of TQ (the main constituent of the volatile oil of black seeds) at three doses (30, 60, and 90 mg/Kg/Day) during 3 months.

Furthermore, the histological observations on the kidney slices show small effects at the lowest dose, with a significant increase, however, of Bowman's space at 6.4 g/kg leading to empty and atrophied glomeruli at intermediate doses (21 and 33 g/kg), whereas slight changes were observed at 60 g/kg.

Dollah et al. [23] found no renal, functional, or lesional damage after administration of 0.01 g/kg, 0.1 g/kg, and 1 g/kg of* Nigella sativa* powder for 5 weeks, while Zaghlol et al. [25] show possible renal toxicity after administration of NS oil to rats, resulting in necrosis of some proximal and distal tubules with any effect on glomeruli at the dose of 15 mL/kg. However, they observed glomerular injury in the form of degeneration of the tuft of capillaries, ill-defined basement membrane, and destruction of endothelial cells, in addition to tubular necrosis with the dose of 25 mL/kg.

It is noteworthy to mention that other authors [26, 27] have shown the protective effect of NS oil against acute renal injury induced by cisplatin.

On the other hand, since most toxic compounds accumulate in the liver where they are detoxified [28], the evaluation of the function and integrity of the hepatic tissues was investigated by measurement of creatine and hepatic enzymes ALT, AST, and AP [29]. However, ALT is more hepatospecific than AST because it is predominant in the liver, whereas AST is found at equal levels in the liver, heart, muscle, kidney, and lungs [30]. Thus, cellular necrosis or destruction of the hepatic parenchyma or increase in membrane permeability of hepatocytes may lead to the flow of these enzymes and then increase their serum levels [31]. AP is a membrane-bound ubiquitous enzyme, especially in the liver, bile ducts, kidneys, bone, and placenta, and is released unequally depending on the pathological phenomenon [32].

This suggests that the AqE.NS's lowest and highest doses have not caused injury on the liver function considering ALT rates, while the intermediate dose (21 g/kg) induced damage leading to disturbance of its function. It suggests also that AST results obtained may reflect injury on other organs because of the high levels observed.

The histopathological examination of liver cuts shows a minor damage at low doses and evidence of degenerative changes in hepatocytes in particular at the intermediate dose by appearance of hemorrhagic, inflammatory, fibrotic, and necrotic zones, in agreement with the results of ALT observed.

Vahdati-Mashhadian et al. [13] and Dollah et al. [23] reported minor liver damage during their studies and no significant variation in hepatic enzymes but it is noteworthy to notice that the experiment of the first authors only lasted for 2 weeks and the second ones used very low doses of NS. Tennekoon et al. [32] reported a significant increase in *γ*-GT enzymes and ALT, without modification of the activities of the AST enzymes and AP, after the oral administration of an aqueous extract of NS to rats under anaesthesia for 14 days under the dose of 10 ml kg/day.

In the light of our findings, it is possible to say that AqE.NS can show the toxicity when it is used at high concentration (>6.4 g/kg), since it can induce the injury in liver leading to disturbance of its function, but the damage will be less important in kidney.

We also observed that the toxic effect may be more important under the AqE.NS's intermediate dose of 21 g/kg and also may differently manifest itself in many individuals that are exposed to the same dose of toxin, such as the 60 g/kg dose, for which we had the largest number of deaths, 3/5. Paradoxically, for the same dose, we obtained the histological observations on the surviving animals approaching those of animal control and somehow a reduction on hepatic enzymes, showing a kind of adaptation of the mice to the high dose and beginning of restoration of the liver function.

### 4.2. Antidiabetic Study on Rats

The AqE.NS 2 g/Kg dose was chosen upon the basis results of the previous study.

Diabetes was introduced in rats by a single intraperitoneal alloxan (ALX) injection in DM and DNS groups and caused disturbance of carbohydrate metabolism, manifested by hyperglycemia, decreased insulinemia, decreased glycogen in the liver, and perturbation of lipid metabolism by increasing total cholesterol, triglycerides, and LDL-c in sera as well as total lipids and triglycerides in liver.

The ALX acts through a cytotoxic action on pancreatic *β* cells mediated by free radical production and by disruption of free cytosolic Ca^2+^ homeostasis of Langerhans *β* cells, thus generating insulin secretion deficiency rather than insulin resistance [33]. The animals then show severe hyperglycemia, glucosuria, polyphagia hyperlipidemia, polydipsia, and various other symptoms of uncontrolled diabetes [3, 33–35].

Moreover, we found that the injection of alloxan did not induce a weight loss in the concerned groups of rats that could suggest the induction of type 1 diabetes but we rather noticed an increase in weight in diabetic animals. Indeed, the oral administration of 2 g/kg AqE.NS to diabetic animals increased weight in the DNS group during the last month of treatment, which could be explained by the rise of lipogenesis as a sign of the augmentation in insulin secretion, joining thus the observations of Benhaddou-Andaloussi et al. [22] on the antidiabetic effect of an aqueous extract of NS on* Meriones shawi* given alloxan.

Our treatment with AqE.NS also shows a gradual return to euglycemia, followed by an increase in insulin in sera, in agreement with the results of Kanter et al. [36] who induced diabetes by streptozotocin (STZ) and those of Rchid et al. [37] and Benhaddou-Andaloussi et al. [38] who demonstrated in vitro that* N. sativa* extract improved *β* cell proliferation and increased insulin secretion stimulated by glucose, consequently attenuating the progression of diabetes.

The lipid profile of diabetic animals which are under our study was illustrated by an increase in TG, total cholesterol, and LDL-c, in accordance with the results already advanced by Al-Logmani and Zari [39, 40] on STZ-induced diabetic rats treated by NS oil, and the significant decrease in these same lipid parameters that we observed after administration of AqE.NS followed by the increase of HDL-c, which is also in agreement with the findings of other authors [41–43].

Indeed, Fararh et al. [44] observed a significant decrease in serum triglyceride levels in diabetic rats treated by thymoquinone (TQ), Asgary et al. [45] reported that NS significantly decreased low-density lipoprotein (LDL), and Zaoui et al. [46, 47] for their part quantified the decrease in serum cholesterol in normal rats at 15.5% and TG levels at 22%. In humans, several studies on different populations (hypercholesterolemic, healthy, diabetic, hypertensive, menopausal, and overweight) reach the same conclusions as those found in animals [8, 48, 49].

Trying to explain the action of NS, several mechanisms can be advanced according to some authors.

The return to euglycemia could be explained by an increase in the synthesis and translocation of GLUT-4 in the muscle contributing to the reduction of hyperglycemia. Indeed, Benhaddou-Andaloussi et al. [22, 50] report that NS stimulates in vitro the phosphorylation of Acetyl-CoA Carboxylase (ACC), a substrate of lipogenesis, via the signaling pathway of AMPK, in skeletal muscle, and that in vivo it also stimulates the phosphorylation of the muscular and liver ACC and increases the expression of muscle GLUT-4.

The hypoglycemic effect can also be explained by a reduction in gluconeogenesis in the liver according to Fararh et al. [51] or, as shown by our results, by an improvement in glycogenogenesis.

Although the pathways of this observed hypolipidemic effect have not yet been elucidated, many hypotheses are nevertheless advanced, such as the action of certain NS components like thymoquinone (TQ) which, in vitro, regulates the genes involved in cholesterol metabolism [52] or the improvement of the PPAR-*α*/RXR *α* expressions evoked by Haas et al. [53] in their in vitro study of HepG2 hepatocytes and Caco-2 intestinal cells, where extracts of NS increased secretion of apolipoprotein A1. Similarly, inhibition of de novo cholesterol synthesis by reducing the hepatic activity of HMG-CoA reductase and stimulating the excretion of bile acids, which contribute to the reduction of serum cholesterol levels, also represents mechanisms for explaining the lipid-lowering effect of the plant. In addition, some authors involve antioxidant mechanisms [42, 54–58].

In fact, this lipid-lowering effect obtained in the above-mentioned studies with various extracts, oil or TQ, does not appear to be due to a single component but rather to the synergistic action of the different components of* Nigella sativa*, like thymoquinone, its various sterols, its flavonoids, and its rich content of polyunsaturated fatty acids.

Moreover, the large increase in HDL-c, as well as the considerable decrease in LDL-c, we obtained in our results, confirmed by lipoprotein electrophoresis, suggests that the administration of NS would have a positive effect on the reversal of cholesterol on one hand and a negative action on the endogenous pathway on the other hand. That is because the increase in HDL-c is in favour of a greater efflux of cholesterol by the peripheral cells, especially the vascular cells, whereas the decrease in LDL-c appears to indicate a decrease in the transport of cholesterol to the peripheral cells or a lesser synthesis of the hepatic VLDLs, thus showing its antiatherogenic and cardioprotective properties, justifying the decrease observed in lipids and triglycerides in liver tissue.

Moreover, an oxidative stress has been demonstrated in type 1 or 2 diabetes by many authors. Therefore, chronic hyperglycemia in diabetes mellitus leads to an oxidative stress created by an imbalance between prooxidant and antioxidant components. This favours glycation reactions which lead to advanced glycation end products. These latter also participate in the developement of the oxidative stress and the inflammatory process. Increased oxidation of cell targets and/or decreased antioxidant defence systems [59] have thus been observed in humans. Besides, the concentration of TBARS, in serum or plasma, is still high in diabetics compared to normoglycemic controls [60, 61].

We have also noted in our study an increase in TBARS serum in diabetic rats, confirming the relation between ALX-induced hyperglycemia and oxidative stress. Results of TBARS obtained after treatment were in favour of an antioxidant effect of NS, joining the findings of Leong et al. [62] who reported a reduction in the level of MDA in the rat after administration of TQ.

Our results also showed an increase in TBARS in the liver (13.7%), adipose tissue (32.25%), and pancreas (30.66%). This latter, as reported by Mansi [63], is a target organ of ALX which has a cytotoxic action by ROS, since alloxan and dialuric acid, a product of its reduction, induce the formation of superoxide radicals and then cause DNA damage, which stimulates poly ADP-ribosylation, a process participating in DNA repair [64].

At the eighth week of treatment, AqE.NS has lowered the rate of the hepatic TBARS joining thus the concentration observed in the control group. This showed a decrease in the hepatotoxicity effect of alloxan by our extract. The same decrease was also observed in pancreatic TBARS. These protective effects may be attributed to the antioxidant properties of the* N. sativa *aqueous extract, which inhibited lipid peroxidation. Our results are in agreement with those of Abdelmeguid et al. [1] who found, in rats treated with aqueous extract and TQ, a significant decrease in pancreatic TBARS with partial regeneration of *β* cells of Langerhans islet, specifying that, ultrastructurally, the aqueous extract prevented cytoplasmic vacuolation and fragmentation of mitochondria and increased the number of secretory granules.

## 5. Conclusion

The aqueous extract of* Nigella sativa* shows its safety at low doses but it has evident hepatotoxicity at a very high dose (21 g/kg), leading to a high mortality rate at 60 g/kg. However, we have noted that the hepatotoxicity caused by alloxan was corrected by AqE.NS, which also indicates a hepatoprotective effect when toxicity is induced.

The dose of 2 g/Kg has showed a significant hypoglycemic effect explained by restoration of insulinemia, an increase of glycogenogenesis, and a very interesting hypolipidemic and antioxidant effect in ALX-induced diabetic rats, which suggests that* Nigella sativa* may be interesting adjuvant support in diabetes therapy and in treating dyslipidemia and coronary heart disease.

## Figures and Tables

**Table 1:** Effect of different doses of the AqE.NS on the survival of mice.

Dose in g of AqE.NS/kg of body weight	Number of dead mice	Latency	Mortality rate (%)
0	0/5	—	0
2	0/5	—	0
6.4	1/5	After 2 weeks	20
21	2/5	After 5 weeks	40
33	0/5	—	0
60	3/5	Respectively, after 3, 4, and 5 weeks	60

**Table 2:** Urea, creatine, albumin, AST, ALT, and AP concentrations of mice.

Parameters	Control (water)	Concentrations of AqE.NS [g/kg]					Chi-square test	
		2	6.4	21	33	60	*χ* ^2^	*p*
Urea (mmol/L)	4.9	4.6	5.2	6.5	6.1	8.6	3.65	0.456
Creatine (*μ*mol/L)	12	7	16	15	16	21	12.25	0.016
Albumin (g/L)	39.6	33.4	41.5	40.7	41.8	42.1	1.37	0.849
AST (U/L)	172.3	261.8	266.6	322.6	267.5	505.1	924.62	<0.001
ALT (U/L)	94.9	96.4	109.85	188.1	104.5	148.8	125.49	<0.001
Alkaline phosphatase (U/L)	110	93	105	93	114	87	10.44	0.034

*p* < 0.05: statistically significant.

**Table 3:** Variation in serum biochemical parameters in *Rattus norvegicus. *Effect of AqE.NS (2 g/kg) after induction of diabetes with alloxan.

	T0	W3	W4t	W8t
Glycemia (mg/dL)				
C	96.39 ± 5.95	81.75 ± 6.95	96.75 ± 4.7	104.75 ± 3.97
DM	120.23 ± 11.56	153.81 ± 7.6	259.55 ± 4.82	258.33 ± 42.31
DNS	97.72 ± 13.76	150.07 ± 7.67	107.17 ± 6.02	106 ± 4.24
Triglycerides (mg/dL)				
C	95.73 ± 12.78	118.72 ± 7.08	145.18 ± 17.6	81.68 ± 0,46
DM	98.01 ± 21.25	141.61 ± 6,47	157.23 ± 10.41	157.28 ± 1.9
DNS	99.84 ± 22.66	133.22 ± 9.58	108.06 ± 24.79	75.53 ± 3.5
Total cholesterol (mg/dL)				
C	107.42 ± 24.58	70.96 ± 9.84	54.57 ± 2.40	60,86 ± 0.69
DM	68.52 ± 9.28	122.06 ± 30.99	98.41 ± 4.04	114.61 ± 7.91
DNS	120.24 ± 29.40	151.79 ± 21.78	72.51 ± 9.42	72.41 ± 7.78
HDL-c (mmol/L)				
C	1.22 ± 0.07	1.25 ± 0.06	0.99 ± 0.06	1.13 ± 0.01
DM	1.22 ± 0.33	1.39 ± 0.18	1.33 ± 0.18	1.48 ± 0.07
DNS	1.1 ± 0.08	1.2 ± 0.13	1.43 ± 0.09	1.68 ± 0.02
LDL-c (mmol/L)				
C	0.86 ± 0.28	0.47 ± 0.11	0.7 ± 0.06	0.95 ± 0.15
DM	0.41 ± 0.05	1.34 ± 0.07	1.25 ± 0.05	1.40 ± 0.03
DNS	0.67 ± 0.25	1.67 ± 0.1	0.27 ± 0.08	0.33 ± 0.09
Insulinemia (UI)				
C	37.77 ± 2	31.99 ± 4.17	-	32.15 ± 3.98
DM	39.39 ± 4.06	29.42 ± 7.55	-	25.49 ± 1.61
DNS	39.13 ± 2.35	25.74 ± 4.60	-	34.50 ± 3.73
AST (U/L)				
C	136.1 ± 11.45	127.53 ± 13.62	121.48 ± 1.76	147.7 ± 32.54
DM	102.4 ± 0.65	140.97 ± 22.19	138.27 ± 34.33	128.5 ± 11.65
DNS	102.48 ± 0.51	110.5 ± 8.88	108.73 ± 12.58	137.43 ± 16.47
ALT (U/L)				
C	63.4 ± 2.25	52.83 ± 6.22	64.65 ± 3.14	60.01 ± 0,73
DM	53.8 ± 6.36	67.3 ± 11.21	72.17 ± 2.52	78.81 ± 4.36
DNS	56.7 ± 3.71	66.2 ± 5.21	37.85 ± 1.43	40.35 ± 1,23
Sera TBARS (*µ*M/L)				
C	9.05 ± 2.97	6.45 ± 2	-	4.95 ± 0.36
DM	5.63 ± 1.31	9.85 ± 2.33	10.75 ± 2.48	11.8 ± 1.1
DNS	4.35 ± 0.59	8.1 ± 0.34	8.09 ± 1.14	4.94 ± 0.14
Erythrocytic TBARS (*µ*M/L)				
C	13.38 ± 0.53	9.38 ± 1.02	14.35 ± 3.38	11.31 ± 4.92
DM	12.48 ± 9.16	8.77 ± 1.11	14.78 ± 5.27	16 ± 3.01
DNS	12.00 ± 1.48	12.06 ± 1.64	9.40 ± 1.96	7.9 ± 0.65

Values are expressed as means ± SM. C: control group; DM: diabetic rats model; DNS: diabetic rats treated with 2 g AqE.NS/Kg body weight; T0: initial time of experiment; W3: 3 weeks after injection of alloxan; W1t to W8t: weeks of treatment by AqE.NS after induction of diabetes in the third group, DNS.

**Table 4:** Two-way ANOVAs testing the effects of rat treatments: “Groups,” treatment duration “Time,” and their interaction “Groups × Time” on the variation of serum biochemical parameters in *Rattus norvegicus.*

Parameters	Groups of rats		Time		Groups × Time	
	*F*	*p*	*F*	*p*	*F*	*p*
Glycemia	76.56	<0.001	11.94	<0.001	12.67	<0.001
Triglycerides	6.35	0.005	6.07	0.002	2.36	0.056
T-Cholesterol	5.18	0.012	4.10	0.015	3.54	0.009
HDL-c	5.87	0.008	3.16	0.041	3.15	0.018
LDL-c	11.39	<0.001	11.25	<0.001	21.12	<0.001
Insulinemia	0.29	0.749	5.05	0.013	0.90	0.477
TBARS in sera	1.29	0.291	7.99	0.001	1.95	0.108
TBARS in erythrocytes	0.96	0.394	0.72	0.549	1.19	0.341
AST	1.75	0.190	1.32	0.287	0.82	0.565
ALT	16.01	<0.001	0.55	0.650	8.18	<0.001

*F*: *F*-statistics; *p*: *p* value. Difference is significant when *p* < 0.05.

**Table 5:** Two-way ANOVAs testing the effects of rat treatments: “Groups,” treatment duration “Time,” and their interaction “Groups × Time” on the variation of weight of *Rattus norvegicus*.

Parameter	Group		Time		Group × Time	
	*F*	*p*	*F*	*p*	*F*	*p*
Weight	97.42	<0.0001	9.25	<0.0001	0.80	0.7148

*F*: *F*-statistics; *p*: *p* value. Difference is significant when *p* < 0.05.

**Table 6:** Changes of the TBARS in tissues (liver, pancreas, and adipose tissue) in *Rattus norvegicus* after induction of diabetes and treatment with AqE.NS.

TBARS (*µ*M/100 g of tissue)	C	DM	DNS	ANOVA	
				*F*	*p*
Liver	149.16 ± 8.31	169.59 ± 27	152.1 ± 12.01	0.66	0.542
Pancreas	121.41 ± 8.69	158.63 ± 9.8	139.52 ± 12.99	3.70	0.073
Adipose tissue	102.01 ± 26.82	134.91 ± 21.72	133.21 ± 22	0.82	0.475

Values are expressed as means ± SM. *F*: *F*-statistics; *p*: *p* value. Difference is significant when *p* < 0.05. C: control group; DM: diabetic rats model; DNS: diabetic rats treated with 2 g AqE.NS/Kg body weight.

**Table 7:** Evaluation of glycogen, total lipids, and triglycerides in hepatic tissue after 8 weeks of treatment with AqE.NS.

Hepatic tissue (mg/100 g)	C	DM	DNS	ANOVA	
				*F *	*p*
Glycogen	1800.92 ± 235.64	1318.57 ± 126.49	1653 ± 191.83	2.11	0.192
Total lipids	1713.21 ± 110.22	2450 ± 411.23	1562.38 ± 115.53	6.82	0.023
Triglycerides	296.05 ± 31.81	400.88 ± 36.55	318.57 ± 36.49	2.22	0.179

Values are expressed as means ± SM. *F*: *F*-statistics; *p*: *p* value. Difference is significant when *p* < 0.05. C: control group; DM: diabetic rats model; DNS: diabetic rats treated with 2 g AqE.NS/Kg body weight.
